# A diverse diapsid tooth assemblage from the Early Triassic (Driefontein locality, South Africa) records the recovery of diapsids following the end-Permian mass extinction

**DOI:** 10.1371/journal.pone.0285111

**Published:** 2023-05-01

**Authors:** Devin K. Hoffman, John P. Hancox, Sterling J. Nesbitt

**Affiliations:** 1 Department of Geosciences, Virginia Tech, Blacksburg, VA, United States of America; 2 Evolutionary Studies Institute, University of the Witwatersrand, Johannesburg, South Africa; Griffith University, AUSTRALIA

## Abstract

Mass extinctions change the trajectory of evolution and restructure ecosystems. The largest mass extinction, the end-Permian, is a particularly interesting case due to the hypothesized delay in the recovery of global ecosystems, where total trophic level recovery is not thought to have occurred until 5–9 million years after the extinction event. Diapsids, especially archosauromorphs, play an important role in this recovery, filling niches left vacant by therapsids and anapsids. However, the nature of lineage and ecological diversification of diapsids is obscured by the limited number of continuous, well-dated stratigraphic sections at the Permian-Triassic boundary and continuing through the first half of the Triassic. The Karoo Basin of South Africa is one such record, and particularly the late Early Triassic (Olenekian) Driefontein locality fills this gap in the diapsid fossil record. We collected a total of 102 teeth of which 81 are identified as diapsids and the remaining 21 as identified as temnospondyls. From the sample, seven distinct tooth morphotypes of diapsids are recognized, six of which are new to the locality. We used a combination of linear measurements, 3D geomorphometrics, and nMDS ordination to compare these morphotypes and made inferences about their possible diets. Although the morphotypes are readily differentiated in nMDS, the overall morphological disparity is low, and we infer five morphotypes are faunivorous with the other two potentially omnivorous or piscivorous based on their morphological similarities with dentitions from extant diapsids, demonstrating an unsampled taxonomic and ecological diversity of diapsids in the Early Triassic based on teeth. Although ecological specialization at Driefontein may be low, it records a diversity of diapsid taxa, specifically of archosauromorph lineages.

## Introduction

The end-Permian mass extinction event was the largest in Earth history, resulting in the extinction of between 80–96% of marine species [1–4] and approximately 70% of terrestrial species [3, 5]. One of the unique aspects of the end-Permian mass extinction is the apparent delayed recovery of both marine and terrestrial ecosystems right after the extinction interval [3, 6–10]. Global climate and ecosystems were destabilized and remained unstable throughout the Early Triassic [6–8, 11, 12]. As a result, terrestrial vertebrate diversity does not return to pre-extinction levels until the Middle Triassic, possibly even the Late Triassic [8, 13, 14].

A key clade for the Triassic terrestrial recovery is Archosauromorpha (taxa more closely related to crocodylians and birds than lepidosaurs). These reptiles filled ecological niches left open by the extinction of therapsids and parareptiles, including large-bodied carnivorous and herbivorous roles [15]. However, the timing and pattern of this recovery is still debated as phylogenetic hypotheses [16, 17] and continued fossil discoveries [16, 18, 19] place the origin of ecologically and anatomically disparate archosauromorph clades in the Early Triassic or earlier. This is despite the delayed Triassic recovery, where “recovery” is complete, with a stable trophic system [20, 21]. This disparity in lineage versus ecological diversification in archosauromorphs is complicated by limited radioistopic dates in the Early to Middle Triassic, and the limited number of basins that preserve terrestrial records of the end-Permian extinction through Late Triassic recovery [15]. Reconstructing this pattern in archosauromorphs requires sampling throughout the entire Early and Middle Triassic.

The Karoo Basin of South Africa provides one of the best terrestrial records from the Late Permian and Triassic Periods [10, 15, 22]. This basin provides a key window into the terrestrial biotic response and recovery from the end-Permian mass extinction event [9, 10]. The archosauromorph fossil record has been sampled from the Karoo Basin since the early 20^th^ Century [23]. Sampling within the Karoo Basin is patchy throughout the stratigraphic sequence and a crucial gap is in the late Early Triassic paleoecosystems (= *Langbergia*-*Garjainia* Subzone [24]) which inhibits reliable models of recovery [9]. The late Early Triassic (Olenekian) Driefontein locality is the best stratigraphic section for the study of tetrapod recovery in this interval. Here, our study exemplifies the importance of this locality in terms of Early Triassic recovery by critically examining the numerous isolated teeth. We identified several morphotypes from this locality which were previously unknown and our study further highlights the rich diversification of varied archosauromorphs and temnospondyls after the end Permian extinction event, providing both taxonomic and ecological information [14, 25–27].

### Locality

Geologically, the Early Triassic (late Olenekian) Driefontein 11 locality occurs in the lower part of the Burgersdorp Formation (Takastad Subgroup, Karoo Supergroup) of South Africa. This locality is the stratotype for the recently proposed *Langbergia*-*Garjainia* Subzone of the *Cynognathus* Assemblage Zone [24]. The *Langbergia*-*Garjainia* fauna of the Driefontein locality correlates with the *Parotosuchus* Fauna of Russia [24], which itself is correlated with Early Triassic (late Olenekian) ammonite-bearing fauna of western Kazakhstan and the Cis-Caspian depression [28].

Currently, the Driefontein locality is known to preserve freshwater chondrichthyans (*Lissodus tumidoclavus*, *Polyacrodus*), actinopterygians (*Saurichthys*), and sarcopterygians (*Ptychoceratodus phillipsi*) [24, 29]. The tetrapod record includes temnospondyl amphibians (*Bathignathus poikilops* [30], *Kestrosaurus dreyeri* [31], *Kestrosaurus kitchingi* [31], *Parotosuchus haughtoni* [32], *Trematosuchus sobeyi* [33]), the therocephalian *Microgomphodon oligocynus* [34], and cynodonts (*Cynognathus crateronotus* and *Langbergia modisei* [35]). The locality has also yielded fragmentary remains including eucynodonts [36], indeterminate procolophonids [24], the enigmatic diapsid reptile *Palacrodon browni* [37, 38], and the erythrosuchid archosauriform *Garjainia madiba* [39]. Despite the rich vertebrate diversity at Driefontein, diapsids are relatively rare, but are represented by isolated bones and teeth.

## Materials and methods

**Fig 1:**
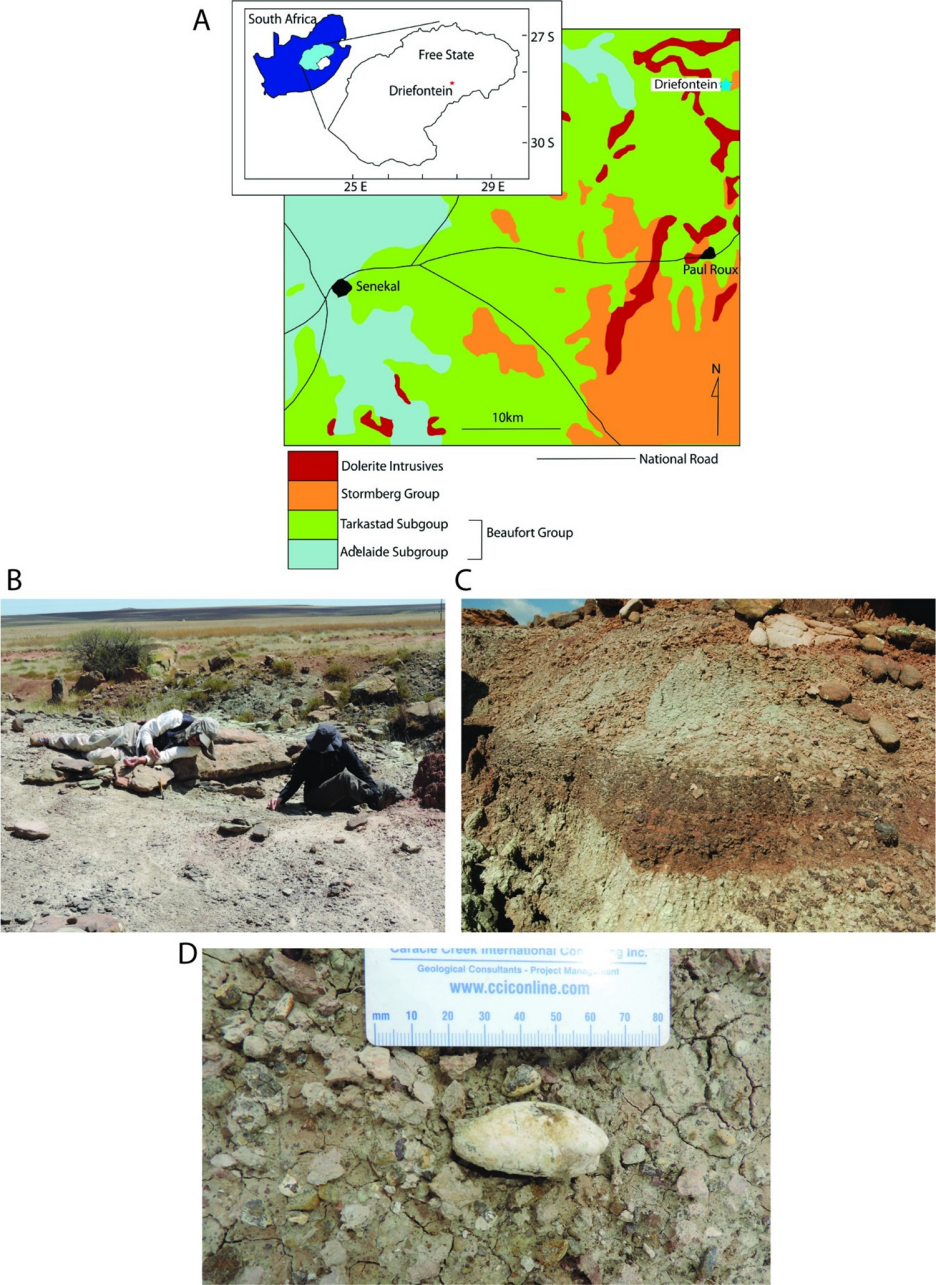
Driefontein locality map and photographs. (A) Simplified geologic map of Driefontein area with inset of location within South Africa. Locality of Driefontein indicated by star. (B) Surface collection of microvertebrate fossils at Driefontein locality. (C) Iron-rich lag channel (in center) which contains richest abundance of fossil material. (D) typical surface lag in field with large coprolite.

**Fig 2:**
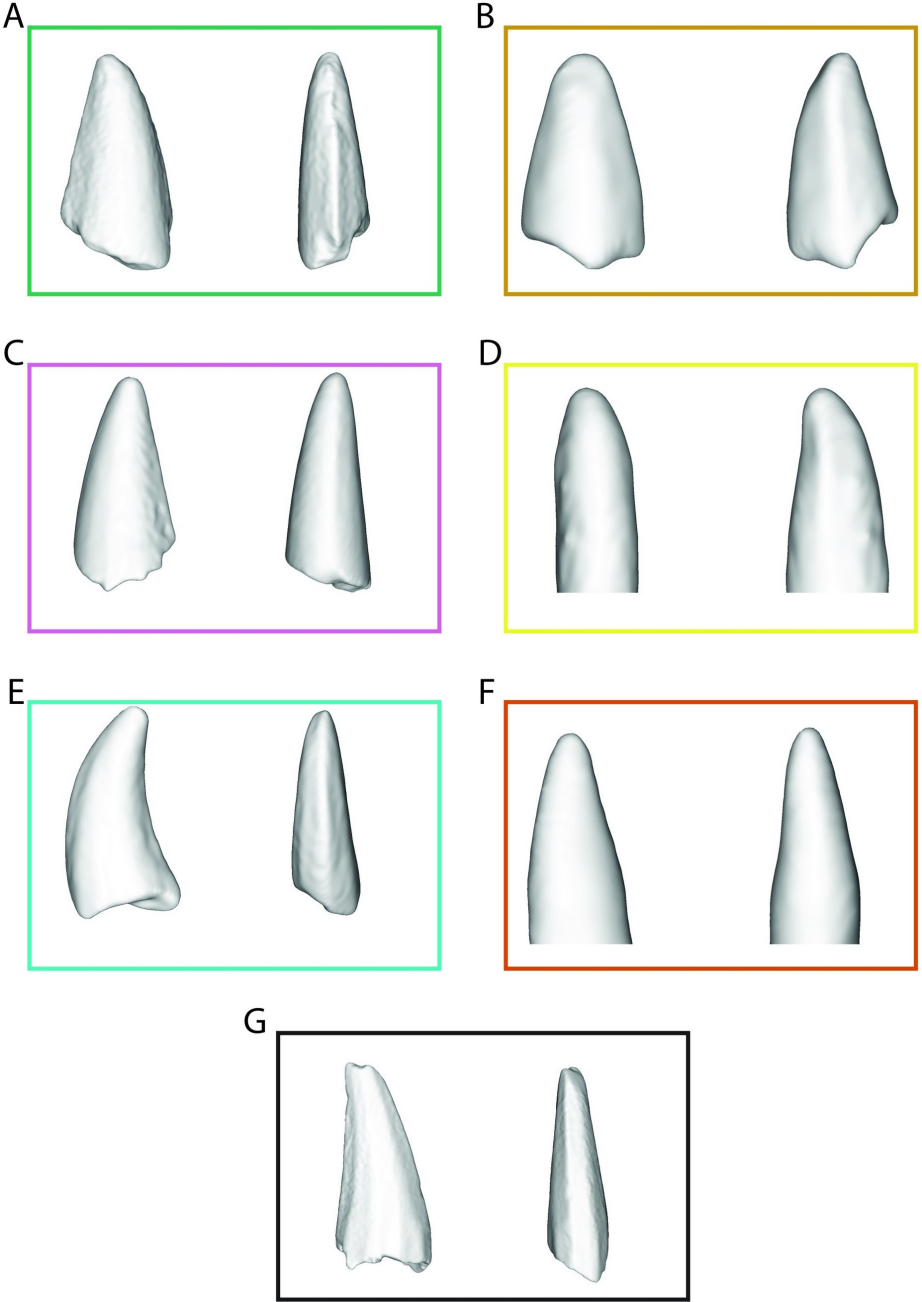
Examples of isolated teeth from Driefontein locality divided by morphotypes. (A) Morphotype A specimen (A382) in lateral and mesial views. (B) Morphotype B specimen (B480) in lateral and mesial views. (C) Morphotype C specimen (F970) in lateral and mesial views. (D) Morphotype D specimen (A660) in lateral and mesial views. (E) Morphotype E specimen (A117) in lateral and mesial views. (F) Morphotype F specimen (E431) in lateral and mesial views. (G) Morphotype G specimen (I084) in lateral and mesial views. Colors correspond to graphics in Figs 4–11. All models made from CT data.

**Fig 3:**
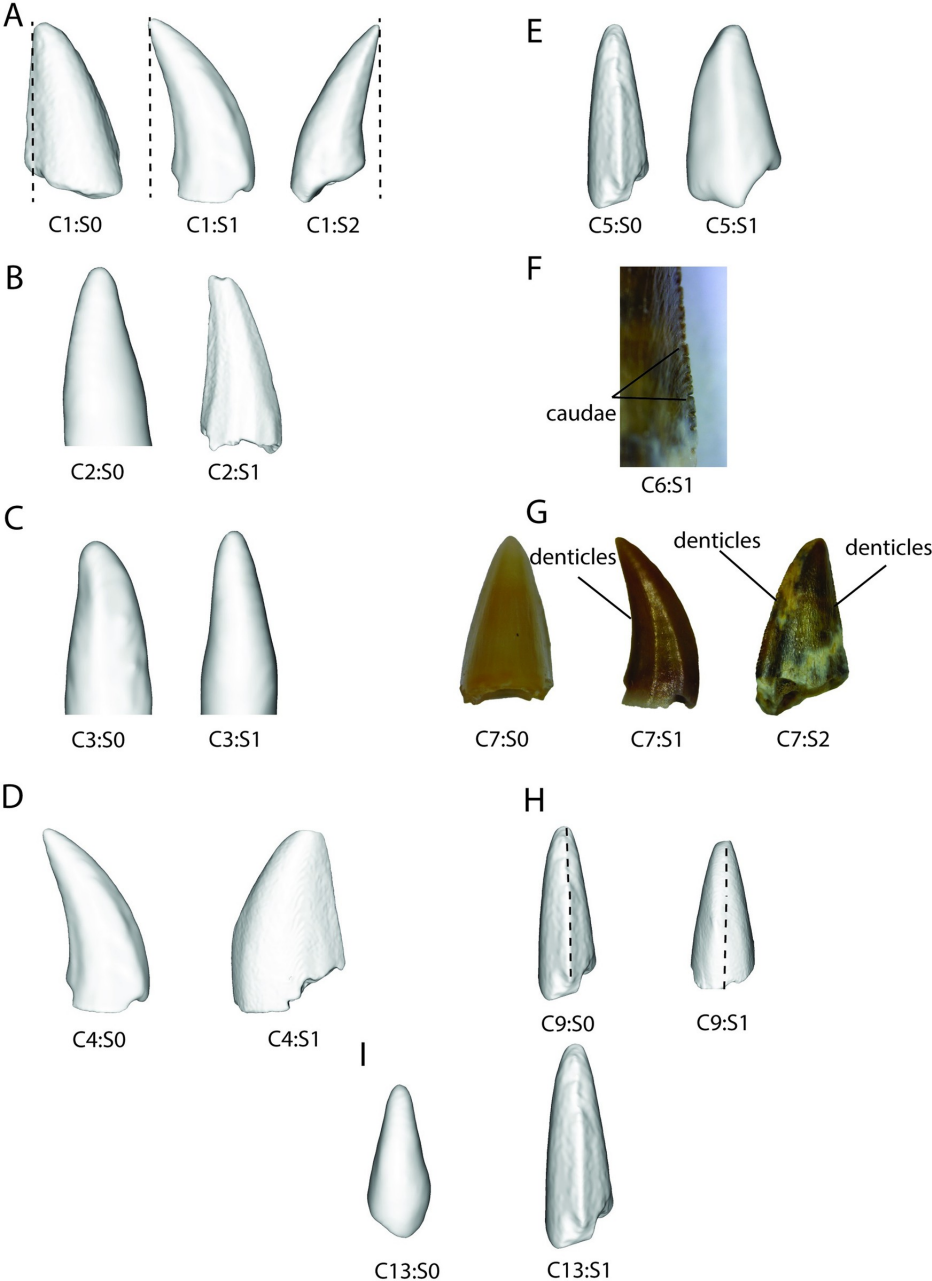
Visualization of traits used in discrete character analysis nMDS. Examples of teeth with different character states, see Table 1 for full descriptions of traits, CX indicates character number, SX indicates state number. (A) recurvedness point above base (left), recurved distally (center), recurved lingually (right), (B) fluting absent (left), present (right), (C) labio-lingual curvature uneven (left), even (right), (D) continuity of mesial edge continuous (left), abrupt shift (right), (E) bulbous–compressed (left), bulbous (right), (F) presence of dental caudae, (G) location of serrations–none (left), distal (center), mesial and distal (right), (H) position of mesial carina midline (left), offset (right), (I) mesial carina absent (left), present (right). Characters and character states 8, 10–11, 13 not shown as CT reconstruction resolution is too low to accurately portray these characters.

All isolated teeth (BP/1/9102-9194) were collected from outcrops on the farm Driefontein 11 in the Bethlehem District, Free State Province, South Africa by PJH (Figs 1–3). All specimens are permanently reposited at the publicly accessible Bernard Price Institute for Palaeontological Research, Johannesburg, South Africa. The Driefontein locality was collected under permit (ID 2678) through the South African Heritage Resources Agency (SAHRA; https://www.sahra.org.za/) permit issued to the Bernard Price Institute for Palaeontological Research for fossil collecting in the Karoo Supergroup.

The geology of Driefontein 11 can be divided into three sections: a lower horizontally-laminated to massive siltstones and mudstones, a middle sandstone-rich unit, and an upper mudstone and siltstone-dominated unit with interbedded sandstones (>1 m thick) [24]. Between the lower and middle sections are intraformational lag conglomerates [24]. The microvertebrate remains are preserved as fragmentary, disarticulated, multi-taxa assemblages within the intraformational lag conglomerates [24]. Microvertebrate-rich sedimentary samples were collected from these lag deposits at Driefontein 11 using techniques outlined in [40, 41] (Fig 1B). Sediment was then screened through sieves and picked under a light microscope. All the specimens used in the present study are from the Evolutionary Studies Institute (University of the Witwatersrand, Johannesburg, South Africa). Upon onset of this study all specimens were loaned to Virginia Tech and have been returned for permanent repository.

In addition to tooth measurements such as total crown height, basal width, and mesial-distal length (Fig 2), comparative measurements were taken from *Garjainia madiba* (NMQR 3051; BP/1/5525, 6232, 7138, 7153) for comparative analysis [24]. This was done because *Garjainia* is the only archosauriform from Driefontein where teeth are preserved in taxonomically identifiable jaws. Based on morphological features such as the presence of serrations [42, character 168, states 1 & 2] and ziphodont morphology, including labial-lingual compression [43, 44], 35 of the isolated diapsid teeth can be assigned to Archosauriformes. The remaining 46 teeth are identified as indeterminate diapsid teeth [27, 45, 46]. Few teeth (n = 21) are identified as temnospondyl based on the presence of plicidentine and labyrinthine enamel [47].

**Fig 4:**
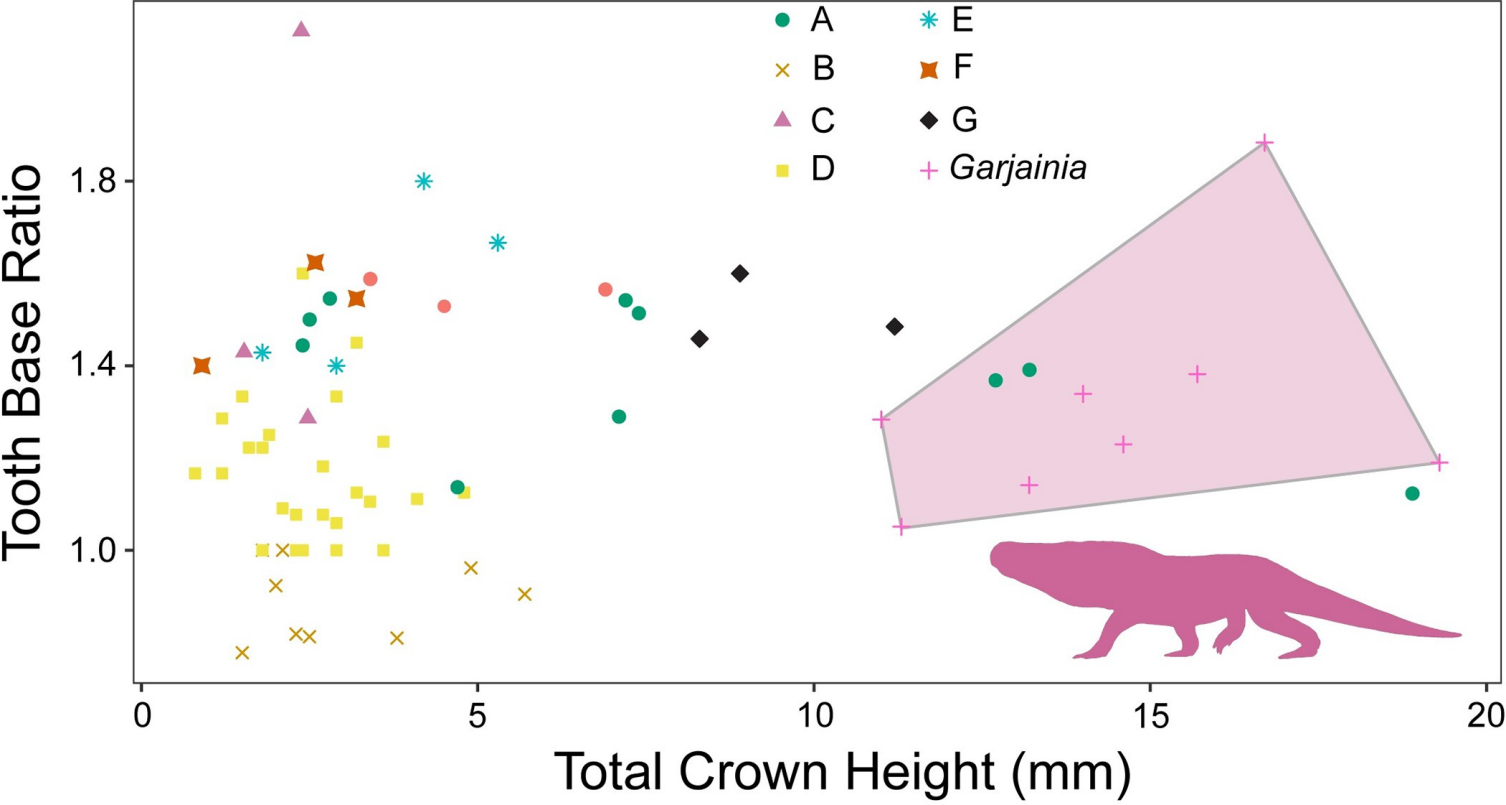
Relationship between tooth height and tooth base ratio. Teeth measurements divided and labeled by morphotype. All the morphotypes have overlapping size and shape distributions. Transparent polygon represents the convex hull of *Garjainia madiba* tooth morphospace, which is only shared by morphotype A teeth. Silhouette represents *Garjainia madiba*.

**Fig 5:**
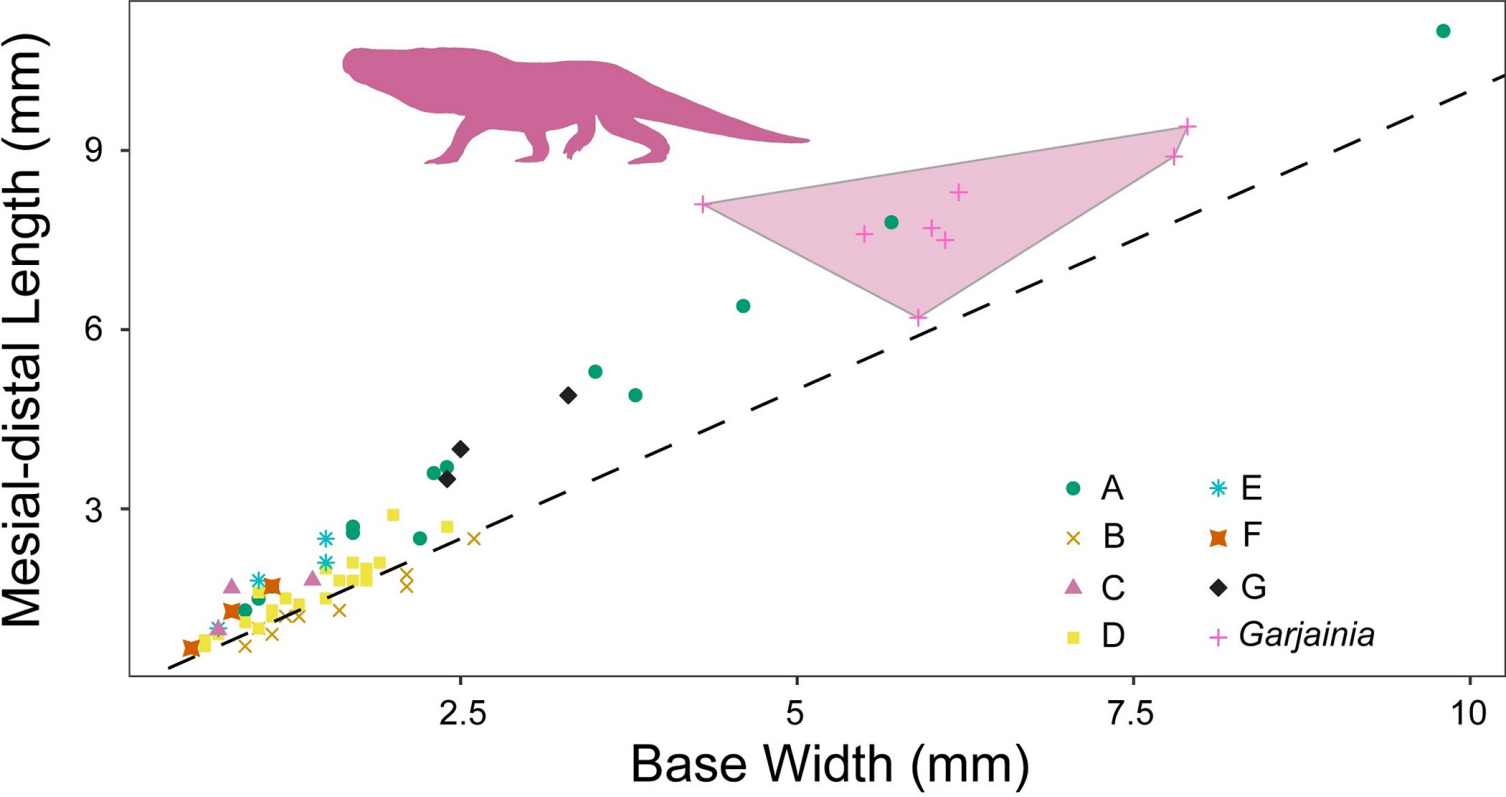
Relationship between tooth base width and mesial-distal length. Teeth measurements divided and labeled by morphotype. All the morphotypes have overlapping size and shape distributions and only morphotype B lies below 1:1 line. Transparent polygon represents the convex hull of *Garjainia madiba* tooth morphospace, which is only shared by morphotype A teeth. Silhouette represents *Garjainia madiba* and dashed black line represents 1:1 line or a perfectly circular tooth base.

**Fig 6:**
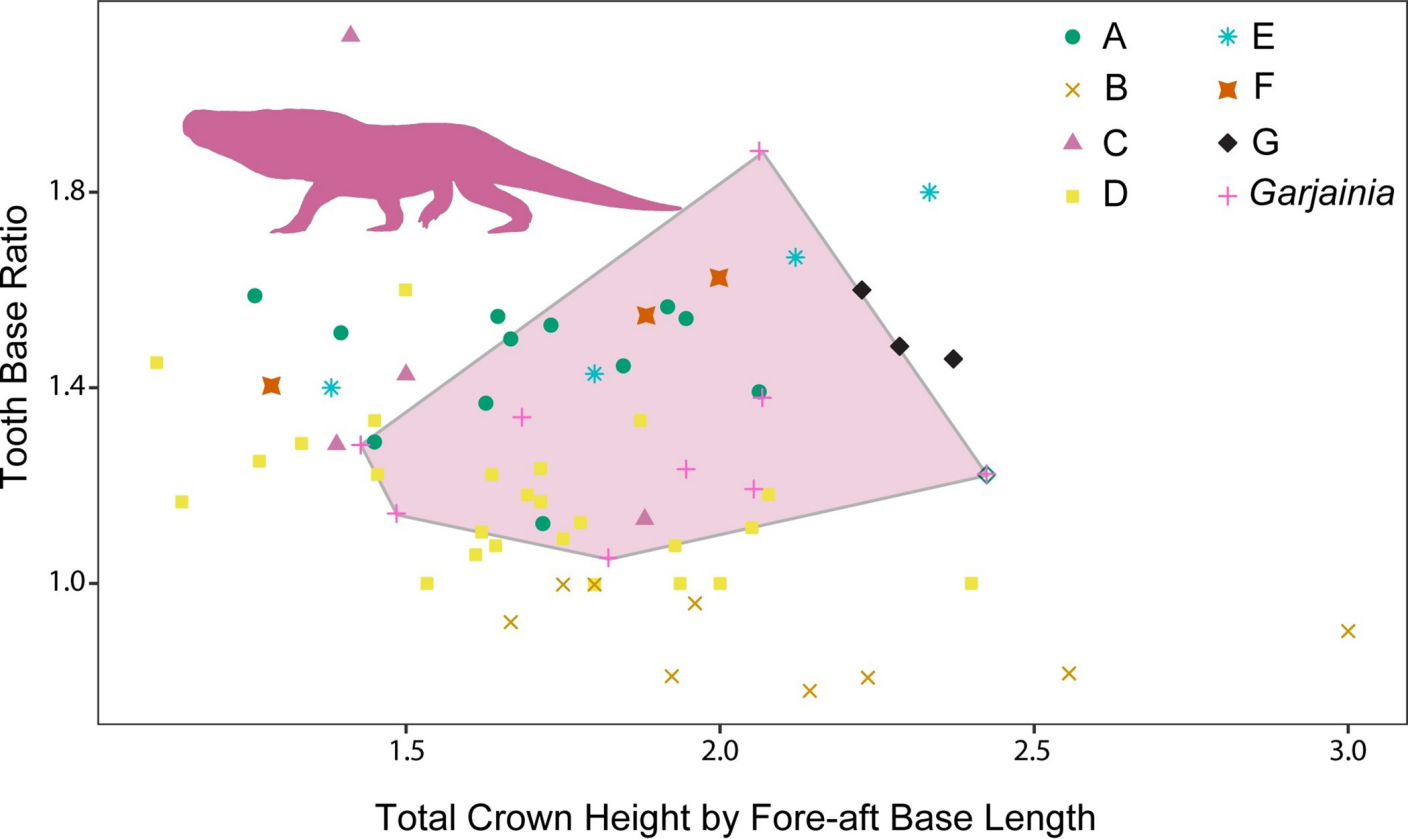
Relationship between tooth height and tooth base ratio controlled for size. Teeth measurements divided and labeled by morphotype. All of the morphotypes have overlapping morphospace with morphotype B being differentiated by a wider than long base (= bulbous). Transparent polygon represents the convex hull of *Garjainia madiba* tooth morphospace and the silhouette represents *Garjainia madiba*.

**Fig 7:**
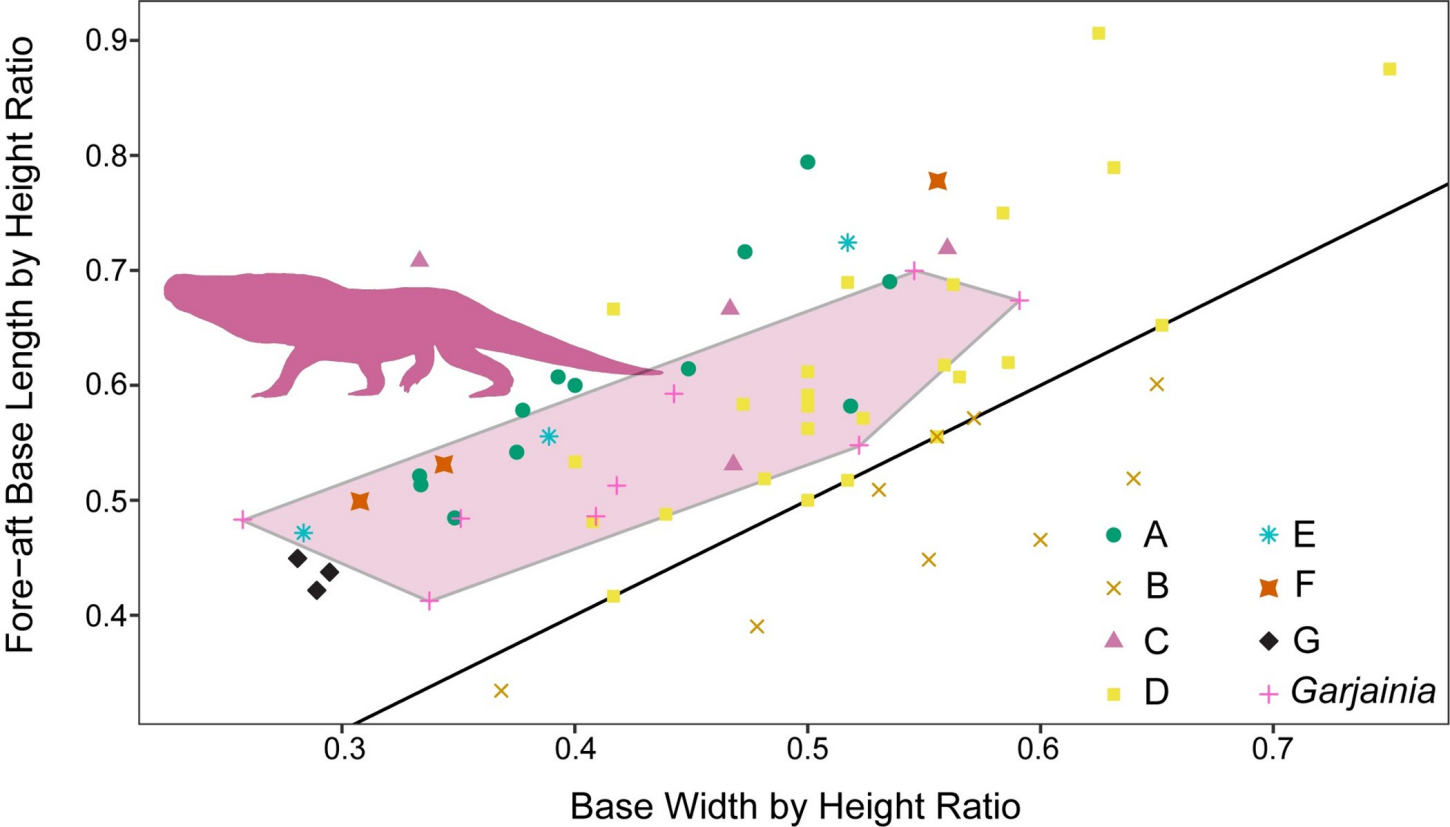
Relationship between tooth base width and length controlled for size. Teeth measurements divided and labeled by morphotype. Overlap between morphotypes is greater than without controlling for size. Transparent polygon represents the convex hull of *Garjainia madiba* tooth morphospace, which is only shared by morphotype A teeth. Silhouette represents *Garjainia madiba* and black line represents 1:1 line or a perfectly circular tooth base.

A total of 102 teeth were used in this study (Figs 2 and 3) and quantitative analyses were performed on 92 of these teeth due to incomplete preservation of 10 teeth. Several parameters such as total crown height (TCH), fore-aft basal length (FABL), basal width (BW) to the nearest tenth mm and denticle densities (denticles per 1 mm) were measured using a digital caliper (data in S1 Dataset). These measurements were used to reconstruct the overall shape and size variation (Figs 4 and 5) of the teeth following [14] using the package ‘ggplot2’ [48] in the R environment (v. 4.1.1) with RStudio (v. 2021.9.1.372). We also calculated differences unrelated to size by measuring different ratios such as total crown height by both fore-aft basal length (Fig 6) and basal width by total crown height (Fig 7).

For the 3D morphometrics, we generated 3D models of 92 individual teeth from μCT scans made using a SkyScan 1172 (Virginia Tech). All scans were conducted with the following settings: medium camera with 34.9 μm pixel, rotation step of 0.9, frame averaging of five, random movement of 10, 360° rotation, an Al + Cu filter, and X-ray with 70 kV voltage, 142 μA current, and 10 W power. The μCT data were transformed into individual mesh models of each tooth using MeshLab [v. 2020.12, 49]. Mesh models were imported into the SlicerMorph module [50] of the platform 3DSlicer [v. 4.11.0, 51] and pseudolandmarks were placed using the PseudoLMGenerator extension [52].

We used the SlicerMorph extension ALPACA [53] to apply the same pseudolandmarks to all other meshes and following [53], we used different template meshes from three different morphotypes to test for bias in template pseudolandmarks. Only complete teeth from Morphotype A (A382), D (A660), and E (A177) were used to test for any effect of template choice. Because there was no visual difference in results, the A177 results were chosen arbitrarily.

**Fig 8:**
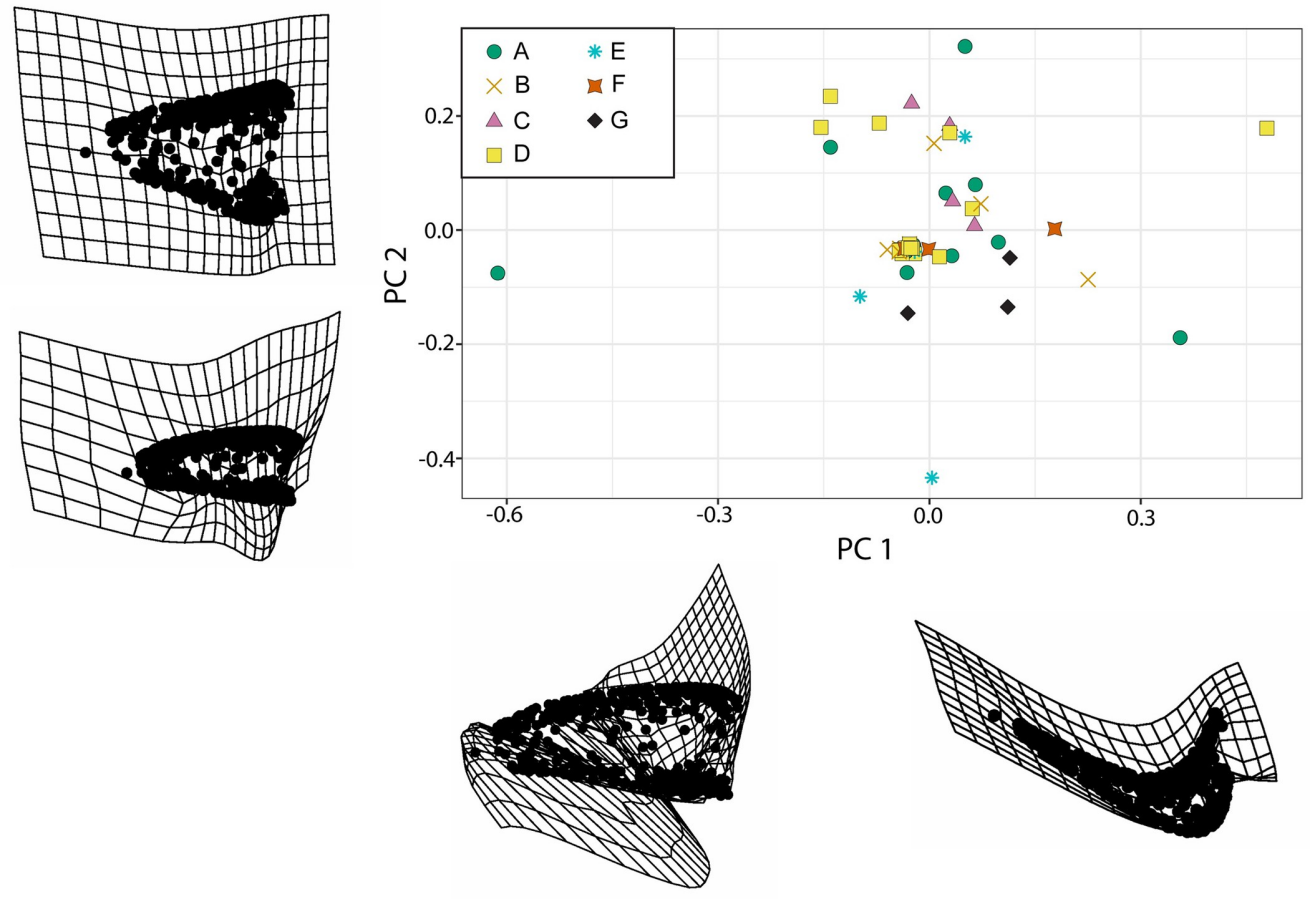
PCA visualization with all complete reptile teeth. Morphospace plotted on PC1 and PC2, each point represents a single tooth. Thin-plate splines for minimum (left) and maximum (right) PC values on the first major axis. Variation in this axis is dominated by recurvedness. Thin-plate splines for minimum (bottom) and maximum (top) PC values on the second major axis. Variation in this axis is dominated by labio-lingual compression.

**Fig 9:**
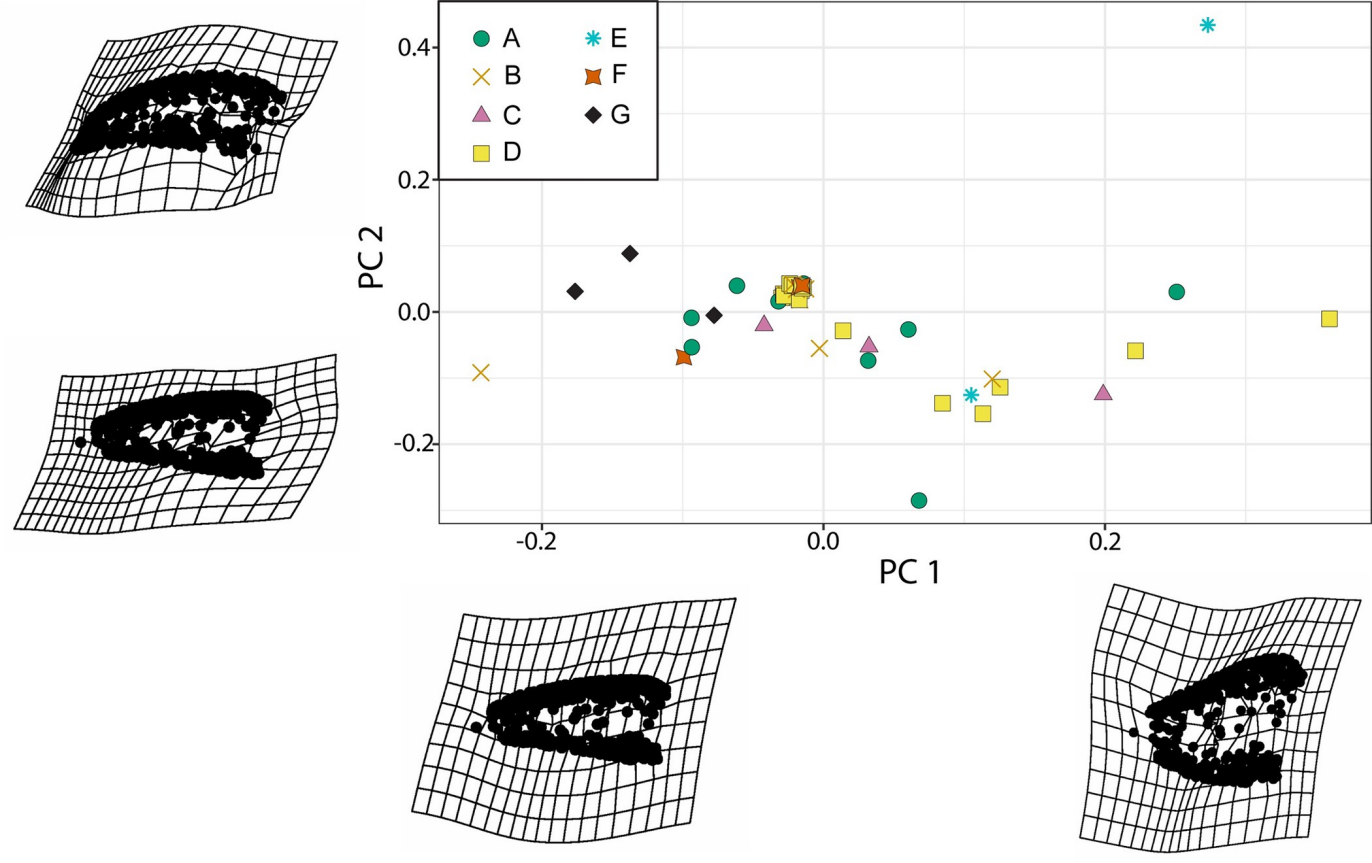
PCA visualization with outliers removed. Morphospace plotted on PC1 and PC2, each point represents a single tooth. Thin-plate splines for minimum (left) and maximum (right) PC values on the first major axis. Variation in this axis is dominated by lateral profile (spade-like versus conical). Thin-plate splines for minimum (bottom) and maximum (top) PC values on the second major axis. Variation in this axis is dominated by recurvedness.

As all results were similar, we only present the results of one template using 321 pseudolandmarks. Due to current incompatibility between ALPACA in SlicerMorph and the R package ‘geomorph’ we used the R package ‘SlicerMorphR’ to convert the pseudolandmark data from SlicerMorph into a usable format. We then conducted PCA using ‘geomorph’ in the R environment (Fig 8), and then a secondary PCA on a modified set of teeth with outliers from the initial PCA removed (Fig 9). This was used to produce thin-plate splines for visualization of Principal Components (PCs) (Figs 8 and 9). Thin-plate splines were chosen to visualize shape as it allows for a two-dimensional representation of landmark variation.

**Fig 10:**
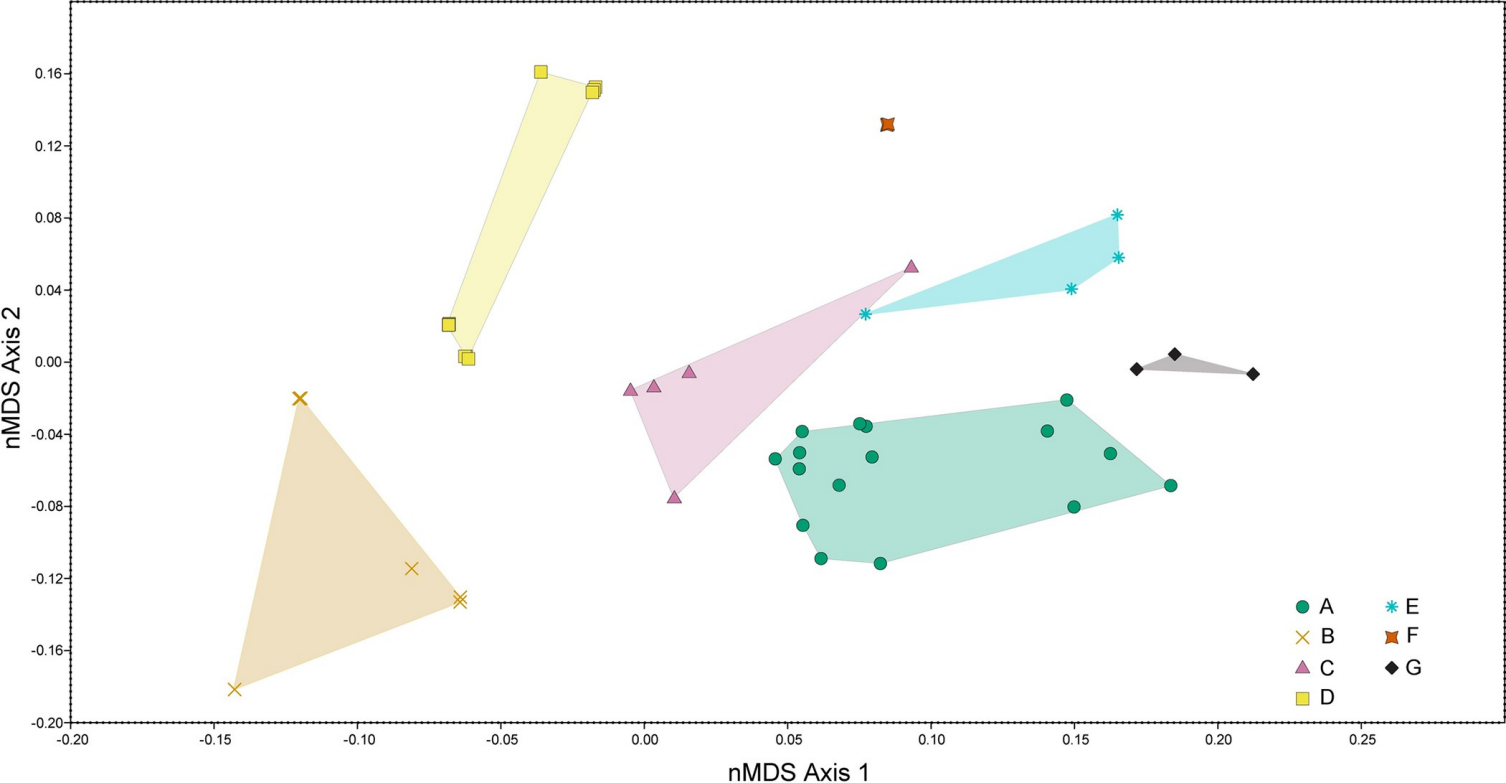
Ordination plot of first two major nMDS axes of reptile tooth morphospace. Colored, transparent polygons are convex hulls of each reptile tooth morphotype, colors and symbols correspond to different morphotypes. None of the convex hulls overlap, indicating that the qualitative scoring for each tooth morphotype is quantitatively distinct. Axis one is mainly divided by serrations with non-serrated teeth having negatively weighted values and serrated teeth having positively weighted values.

**Fig 11:**
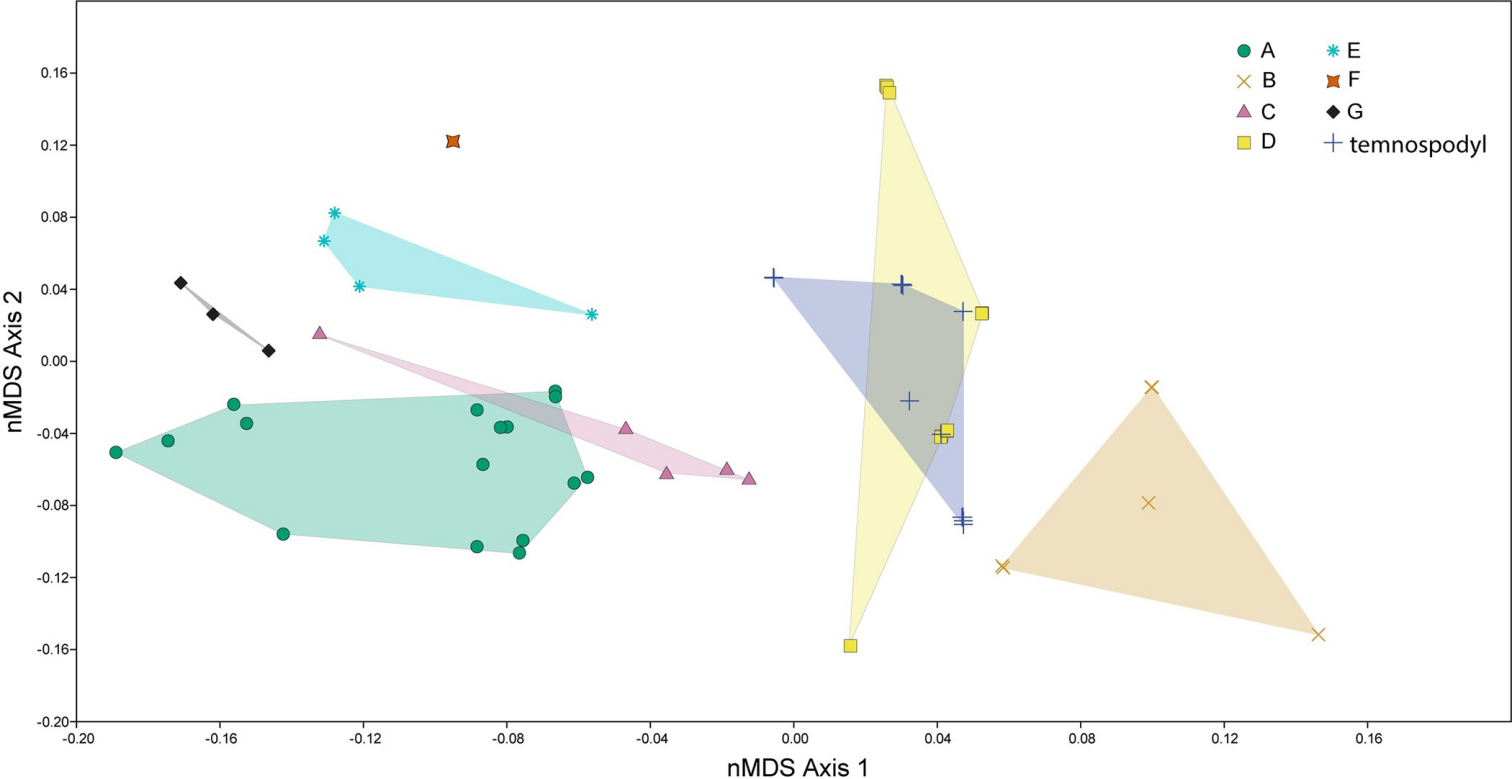
Ordination plot of first two major nMDS axes of diapsid tooth morphospace and temnospondyls. Colored, transparent polygons are convex hulls of each reptile tooth morphotype and the temnospondyl morphotype, colors and symbols correspond to different morphotypes. There is some overlap in morphotypes A and C and between morphotype D and temnospondyl teeth as the new analysis results in new optimal distances. The first major axis is still split by serrations, though now positive weights are non-serrated and negative weights are serrated.

We used nMDS to capture qualitative features of teeth in a quantifiable and reproducible fashion. We created a set of 13 discrete characters, with two or three character states, that capture morphology relevant to our sample of teeth (Table 1, scorings in S2 Dataset). Nine are taken from [14], one from [54], and the remaining three were created for this analysis to capture the variation within our sample (Table 1, Fig 3). We used paleontological statistics [PAST, v. 2.17c, 55] to run the nMDS analysis on 72 isolated reptile teeth using a Bray-Curtis transformation (Fig 10). We ran a new analysis under the same conditions with 21 indeterminate temnospondyl teeth for additional comparisons (Fig 11).

**Table 1:** Summary of discrete, descriptive features of isolated teeth used in nMDS analysis.

	Trait Description
1	Tooth apex, location, relative to the tooth base: tip within or above the edge of the tooth base (0), tip is located more distal than the distal edge (= recurved) (1), or lingual to the lingual most edge of the tooth base (2) [modified from 14]
2	Tooth lingual/labial, surfaces: texture is smooth (lack of crenulations, ridges, etc.) (0) or surface texture possess a series of parallel ridges from tooth apex to base (= fluted) (1) [from 14]
3	Tooth labial/lingual, shape: crown curvature unequal (one side expanded relative to other) (0) or equal labial and lingual curvature (1) [from 14]
4	Mesial tooth margin, shape: curvature angles change gradually (0) or angle changes abruptly at a single discrete point along mesial edge (1) [from 14]
5	Tooth crown, size: labiolingual widths dorsal to the tooth crown base are all less than the crown base width (0) or a crown labiolingual width dorsal to the tooth crown base is greater than the crown base width (1) [from 14]
6	Mesial/distal crown margins, surfaces: denticle caudae (= grooves on crown surface from between individual denticles) are absent (0) or present (1) [from 54]
7	Mesial/distal crown margins, surfaces: no serrations on either mesial or distal surface/carina (0) serrations on distal carina but no serrations on mesial surface (1), or serrations present on both mesial and distal carinae (2)
8	Mesial/distal margins, denticle density: number of mesial and distal denticles is < 6 per mm (0), or greater than or equal to 6 per mm (1). Measurements are taken near the middle of the carina [modified from 14]
9	Mesial margin, location: vertical axis of the mesial carina is in line the mesial-distal long axis (0) or laterally offset from the mesial distal long axis (1) [from 14]
10	Mesial/distal margins, size: average size of mesial and distal denticles are the same (0) or the average size of the mesial and distal denticles is different (1) [from 14]
11	Mesial/distal margins, shape: lateral profile shape of mesial and distal denticles remains constant (0) or denticles’ lateral profile changes shape (e.g., rounded to square) (1) [from 14]
12	Mesial margin, surface: the mesial surface of the tooth is smooth (0) or the mesial surface possesses a carina (1). Note this feature can still be scored (1) even if carina lacks serrations (Trait 7, State 0).
13	Mesial/distal margins, shape: lateral profile shape of mesial or distal denticles (if present) are square (0) or rounded (1)

## Results

### Morphotype A

Within our sample, morphotype A teeth (n = 18, Fig 2A) range in crown height from 2.4 to 18.9 mm, fore-aft basal length from 1.3 to 11 mm, and in basal width from 0.9 to 9.8 mm. These teeth are triangular labio-lingually and few are recurved, but in the other cases within the morphotype, the tip of the tooth is nearly even with the crown base (Figs 2A and 3A). The crown surface lacks apico-basally oriented ridges and valleys (= fluting), and the labial surface is more convex than the lingual, resulting in a curvature (Fig 2A). In some specimens, the mesial edge is gently curved but in others there exists a single point on the mesial edge that abruptly shifts angles (Fig 3D). The tooth base ratio (FABL/BW) is 1.4 such that teeth are labio-lingually compressed. Mesial and distal carinae are serrated (Figs 2A and 3G). In some cases, the mesial carina is offset from the long axis of the tooth base. The distal denticles extend from the tip of the tooth to the crown base, whereas the mesial denticles cover half the crown. The denticles are often square shaped with others rounded and remain the same shape throughout the denticle series. The denticle density varies from 4 per mm in the largest teeth to 12 per mm in the smaller teeth, though the density varies along the carinae. Dental caudae *sensu* [54], shallow grooves between adjacent denticles, are present along the series and oriented perpendicular from the carina.

### Morphotype B

The morphotype B teeth (n = 14) range from 1.1 to 7.8 mm in total crown height, in fore-aft basal length from 0.7 to 3.2 mm, and in basal width from 0.9 to 3.8 mm (Fig 2B). The tooth outline is triangular labio-lingually and in few is hooked, where the tooth tip is lingual to the lingual most edge of the crown base, though in most the tooth tip is even with the distal end of the crown base (Fig 3A). The labial and lingual surfaces of the teeth are covered apically-basally directed ridges and valleys (= fluting) (Fig 3B), and the labial curvature is convex whereas the lingual curvature is concave in mesial view (Fig 2B). Unlike all the other diapsid teeth of the Driefontein locality, morphotype B teeth are bulbous with a tooth base ratio of 0.9 (Fig 2B). These teeth have both a mesial and distal carina but lack any serrations except for a single case (A845), which completely lacks a mesial carina. In most cases the mesial carina is along the fore-aft basal length axis, but there are two specimens where this is offset (Fig 3H).

### Morphotype C

The morphotype C teeth (n = 5) range from 1.5 to 4.7 mm in total crown height, fore-aft basal length from 1 to 2.5 mm, and in basal width from 0.7 to 2.2 mm (Fig 2C). Morphotype C teeth are triangular labio-lingually and are not recurved, although the distal margin is convex in lateral view (Fig 2C). The labial and lingual surfaces are smooth and in all but one case the labial surface exhibits a greater degree of curvature. The fore-aft basal length is greater than the basal width (base ratio = 1.5), resulting in a slight labio-lingual compression. Mesial and distal edges have carinae, but only the distal carina possesses serrations (Fig 2G). Most morphotype C teeth are too poorly preserved for accurate denticle density measurements, but in those that are well preserved, distal denticle densities vary from 9 to 12 denticles per mm. When denticles are preserved they remain a constant size and rounded shape along the denticle series. In some cases, dental caudae are present (Fig 3F).

### Morphotype D

The morphotype D teeth (n = 32) range from 0.8 to 4.8 mm in total crown height, fore-aft basal length from 0.7 to 3.1 mm, and in basal width from 0.6 to 2.5 mm (Fig 2D). These teeth are triangular labio-lingually and distally and the tip of the tooth crown is neither recurved nor hooked (Figs 2D and 3A). The labial and lingual surfaces are smooth, and the labial margin is expanded relative to the lingual margin in mesial or distal view. All teeth are bi-carinate and lack denticles. Unlike morphotype B, the other non-serrated, bi-carinate spade-like tooth morphotype, the fore-aft basal length is always greater than the basal width (base ratio = 1.1) such that the tooth is slightly labio-lingually compressed (Fig 2D). The mesial carina is most often along the long axis of the crown base (Figs 2D and 3H).

### Morphotype E

The morphotype E teeth (n = 5) range in size from 1.8 to 5.3 mm in total crown height, fore-aft basal length from 1 to 2.5 mm, and in basal width from 0.7 to 1.5 mm (Fig 2E). These teeth are triangular labio-lingually and are recurved (Fig 2E). The labial and lingual surfaces bear apically-basally oriented ridges (Fig 3B), and the surfaces are approximately equal in their degree of curvature (Fig 3C). As in most of the morphotypes, the fore-aft basal length is greater than the basal width (Fig 2E), although in morphotype E the degree of labio-lingual compression is more extreme than other Driefontein morphotypes on average (base ratio = 1.6). Morphotype E teeth possess mesial and distal carinae, though only the distal carina has denticles (Fig 3G). The denticle series are poorly preserved in most specimens, but where reliable measurement can be taken denticle density is approximately 10 per mm. The denticles are rounded in lateral view and remain the same shape along the distal carina, although the size of the denticles change, with the largest near the crown base. The mesial carina always forms parallel to the long axis of the crown base (Fig 3H).

### Morphotype F

The morphotype F teeth (n = 3) range in size from 0.9 to 3.2 mm in total crown height, fore-aft basal length from 0.7 to 1.7 mm, and in basal width from 0.5 to 1.1 mm (Fig 2F). These teeth are triangular labio-lingually and are recurved (Fig 2F). The labial and lingual faces of the teeth are smooth, and both the labial and lingual faces have similar amounts of curvature (Fig 2F). The fore-aft basal length is greater than that of the basal width (base ratio = 1.5) such that teeth are laterally compressed (Fig 2F). Morphotype F teeth have mesial and distal carinae but lack denticles on either (Fig 2F). The mesial carina is along the midline of the long axis of the fore-aft basal length (Figs 2F and 3H).

### Morphotype G

The morphotype G teeth (n = 4) range in size from 8.3 to 11.2 mm in total crown height, fore-aft basal length from 3.5 to 4.9 mm, and in basal width from 2.4 to 3.3 mm (Fig 2G). This morphotype is triangular labio-lingually and tends to be recurved (Fig 2G). The labial and lingual surfaces of the teeth bear apically-basally oriented ridges and are equal in their labio-lingual curvatures (Fig 2G). The fore-aft basal length is greater than that of the basal width (base ratio = 1.5), such that morphotype G teeth are laterally compressed (Fig 2G). These teeth have denticle bearing carinae on mesial and distal edges (Fig 3G) with the density of denticles varying from 5 to 8 per mm across the morphotype. Along the crown the denticles remain the same shape and size with square denticles (Fig 3I). The mesial carina parallels the fore-aft basal length long axis (Figs 2G and 3H).

### Remarks

This study found seven distinct diapsid tooth morphotypes from the Driefontein locality (Fig 2). Morphotype A teeth represent 18% of the sample (Fig 2A) and, in all cases, teeth are ziphodont [56] with unequal labio-lingual curvature. This morphotype resembles all known *Garjainia madiba* material; however, we do not refer morphotype A specimens strictly to *Garjainia* because similar tooth morphology is likely plesiomorphic for archosauriforms and is common in carnivorous archosaurs from other Triassic localities, such as *Nundasuchus songeaensis* [14], *Batrachotomus kupferzellensis* [26], and indet. Theropoda(?) [44].

Morphotype B (14% of sample, Fig 2B) resembles morphotypes R19 and R20 of [26] in being conical with a single point, bulbous, and short crown heights. However, R19 lacks the fluting found in morphotypes B and R20. Though not attributable to any particular clade, bulbous teeth with fluting are known in the enigmatic reptile *Colognathus* [57–59] However, the teeth of *Colognathus* are triangular with a much sharper point than any morphotype B teeth in this study.

The teeth of morphotype C (5%, Fig 2C) are the only in the sample with a single set of serrations on the distal carina and smooth surfaces. They share similarities to morphotypes A, E, F, and G, but lacks the mesial serrations of A and G (Figs 2A and 3G), is not recurved like morphotypes F and G (Fig 2F and 2G), and has a smooth surface unlike morphotypes E and G (Fig 2E and 2G). The morphotype P of [60] describes a similar tooth with distal serrations, is labio-lingually compressed, and not recurved [= backswept of 60].

Morphotype D teeth are the most abundant within the sample making up 31% of the included teeth. They superficially resemble the temnospondyl teeth present at the Driefontein locality as well as other Triassic localities [26, 60]. In all cases the teeth are bi-carinate and lack serrations, are generally not recurved, and have a more pronounced labial surface. However, the temnospondyl teeth (n = 21) all possess deep grooves along the labial and lingual surfaces and plicidentine enamel (= labyrinthodont).

Morphotype E teeth (5% of sample) are very similar to those of morphotype C, but differ in possessing apically-basally oriented ridges (= fluting), being recurved, and having equal labial and lingual curvatures (Fig 2E). These teeth resemble morphotype M of [60] in which they are recurved with only distal serrations and a high degree of labio-lingual compression with a base ratio of 1.6, the highest in our sample vs. 1.8 in morphotype M [60]. They differ in that the teeth of morphotype E possess apically-basally oriented ridges and have coarser serrations (10 per mm in morphotype E vs. 25 per mm in morphotype M).

Morphotype F is the least well represented in the study sample (3%) and the only non-serrated, recurved tooth morphotype in the sample (Fig 2F). These teeth are also the smallest of the sample, with a maximum TCH of 1.7 mm, though this could be an artifact of the small sample size. A similar diminutive, recurved, non-serrated tooth morphotype is R16 of [26] which is attributed as a possible lepidosauromorph. However, morphotype R16 lacks carinae, and morphotype F is bicarinate, although the carinae are not as strong as in other morphotypes within the sample such as morphotypes B and D (Fig 2B, 2D and 2F).

Morphotype G teeth make up 4% of the study sample (Fig 2G). Within the study they are most like morphotype A in having mesial and distal serrations and being slightly recurved (Fig 2A and 2G). However, morphotype G has equal labio-lingual curvature and possesses apically-basally oriented ridges (Fig 2G). The morphotype G teeth resemble those of morphotype IX described by [44] which are also ziphodont with fluting and square-shaped denticles. The base of morphotype G teeth are oval, unlike the 8-shaped base of morphotype IX [44].

### Morphometric results

Within this sample, there is much overlap in morphospace occupancy, particularly on the left side of the plot (smaller sizes), with the taller *Garjainia madiba* teeth commonly on the right side of the plot (Figs 4 and 5). The y-axis describes base shape where teeth with high values are more laterally compressed and those closer to one have rounded bases (Fig 4). Only Morphotype B falls below a base ratio value of one with bases wider than long (= bulbous). With crown height alone, three of the Morphotype A teeth fall within the *Garjainia madiba* space (Fig 4), consistent with our descriptive predictions. When we break up the crown base ratio into its components, we see all morphotypes tend to follow a trend along the 1:1 line (Fig 5). Again, the primary separation is based on tooth size with the larger *Garjainia* occupying the upper right quadrant of the plot. Because size appears to be the primary factor dividing morphotypes in the previous plots, we repeated these comparisons while controlling for size. When tooth base ratio is compared to total crown height divided by tooth base length, *Garjainia* is no longer separate from the isolated teeth morphotypes and most differentiation between morphotypes is along the vertical axis (Fig 6). Comparing fore-aft base length divided by total crown height to base width divided by total crown height reduces variation from size, but likewise does not result in easily distinguishable areas within the morphospace (Fig 7). The exception is Morphotype B which consistently lies below the 1:1 line, indicative of a tooth base that is wider than long (= bulbous).

To investigate tooth disparity more fully, we used 3D pseudolandmarks and visualized shape using a PCA plot (Fig 8). The results presented in the main text use a Morphotype E tooth (A177) as the template. The first two principal components (PCs) represent approximately 50% of the total variation in the dataset (PC1 = 27.8% and PC2 = 21.5%) and are the axes we focus on for our results. PC1 seems to primarily capture the location of the tooth tip relative to the tooth base (= recurvedness) with more recurved teeth negatively loaded and straight teeth positively loaded and lateral profile of the teeth with more spade-like teeth negatively loaded and more conical teeth positively loaded (Fig 8). PC2 describes the labio-lingual width relative to mesio-distal length (= robustness), with more robust teeth positively loaded and more gracile teeth negatively loaded (Fig 8). However, the majority of individual teeth cluster in the center of the plot with the morphotypes overlapping. We then excluded the teeth at the edge of the PCA plot as outliers (non-statistical determination) and repeated the same process. The first two PCs still represent almost 50% of the total variation (PC1 = 27.8% and PC2 = 19.3%). However, in this PCA plot, PC1 and PC2 seem to represent the opposite components of PC from the previous analysis. PC1 represents the lateral profile with more spadelike teeth positively loaded and more conical teeth negatively loaded (Fig 9). PC2 then describes the recurvedness of teeth where more positively loaded values are strongly recurved, and negatively loaded values are again straighter (Fig 9). Unlike the previous PCA (Fig 8), in this morphospace plot the individual teeth are more separated (Fig 9). However, there is still no obvious visual way to differentiate between morphotypes due to the high degree of overlap in data points.

The nMDS analyses resulted in clearer differentiation between the morphotypes and less overlap in convex hulls. In the diapsid teeth only analysis, there is no overlap in tooth morphotypes at all (Fig 10). The first major axis (nMDS 1) seems to be recording the presence or absence of serrations and denticles, the two non-serrated morphotypes weighted negatively, with serrated morphotypes weighted positively. The second major axis (nMDS 2) continuum appears to be related to symmetry where the more positively weighted values corresponding to equal labio-lingual curvature and a midline mesial carina, and negatively weighted values corresponding to unequal labio-lingual curvature and an offset mesial carina. When the analysis is re-run with the additional 21 temnospondyl teeth, some of the morphotypes overlap (Fig 11). Specifically, the temnospondyl convex hull almost entirely overlaps Morphotype D’s convex hull and there is minor overlap between Morphotype A and Morphotype C (Fig 11).

The present study describes seven diapsid tooth morphotypes from the Early Triassic Driefontein 11 locality. The large-bodied *Garjainia madiba* teeth occupies the largest areas in the morphospace plots (Figs 4 and 5). Morphotype A is the only isolated tooth morphotype that overlaps with *Garjainia madiba* in 2D linear morphospace with size included (Figs 4 and 5). Given the size range of morphotype A teeth in this study, it is possible that morphotype A represents an ontogenetic series of *G*. *madiba*, which would indicate *G*. *madiba* maintained the same tooth morphology throughout ontogeny. This offset from isolated tooth morphotypes was lost when we controlled for individual tooth size, and the *Garjainia madiba* teeth overlapped with several distinct morphotypes in 2D linear morphospace (Figs 6 and 7). Hull polygons show some overlap between morphotypes except for morphotype B (Figs 4–7) because it has a wide base (base ratio 0.7 to <1) compared to all other morphotypes.

## Discussion

The past two decades have seen a surge in quantitative methods for quantifying tooth morphology, especially with regards to reptile teeth, such as 2D/3D geometric morphometrics [56, 61, 62], OPCR [25, 63], and nMDS [14] (see below). The PCA plots visualize overall trends in shape variation among the entire sample of teeth. Firstly, overall tooth morphology among diapsids is conserved in our sample. Despite using over 300 pseudolandmarks for the tooth meshes there is no clear separation between tooth morphotypes in the PCA plots (Figs 8 and 9). This is likely due to the differences between morphotypes, like serrations or carinae, being too small to be detected at computationally relevant pseudolandmark sampling. Instead, the thin-plate splines (TPS) summarize tooth morphological variation across a spectrum, as used in other tooth assemblage studies [64, 65]. This more accurately represents shape variation and can be used to determine the features of interest in a tooth assemblage. For example, in this study, the recurvedness of teeth was a major driver of variation, which is further supported as an important axis of variation in the nMDS plots.

The tooth morphotypes in this study are recovered as distinct morphospace occupants using nMDS ordination (Fig 10), unlike in previous work using nMDS for other tooth assemblages [14]. This breaks down slightly when indeterminate temnospondyl teeth are included in a separate nMDS analysis (Fig 11). This appears to be the result of the temnospondyl teeth sharing similarities to morphotype D teeth in the discrete character scoring. Regardless, this provides us strong quantitative evidence that our tooth morphotypes are valid assignments and indicates nMDS is the preferred method for testing the validity of morphotype descriptions over purely descriptive identifications of morphotype assignments.

When interpreting possible ecological differentiation between the tooth morphotypes, we use both tooth size and overall morphology. Five of the seven morphotypes are single pointed, labio-lingually compressed, and serrated on either the distal margin or both distal and mesial margins, all indicative of faunivory [26, 27, 66]. The variation among the faunivores is primarily due to size with teeth of *Garjainia madiba* and morphotype A being orders of magnitude larger than the smaller morphotypes C, E, F, and G (Figs 4 and 5).

The other two morphotypes (B and D) lack serrations, recurvedness, and other “hallmarks” of faunivorous diet, though they also lack the complexity associated with herbivory [27]. Therefore, it is difficult to assign these morphotypes to a trophic position, although the similarity to temnospondyl teeth could indicate a piscivorous diet [47]. Morphotypes B and D teeth also resemble the teeth of ichthyosaurs [67], further lending support to the assignment of piscivory.

A single diapsid tooth with coarse serrations that superficially resembles azendohsaurid teeth [68] was found after the initial loan of material and could not be included in the analyses. However, its presence indicates that there were archosauromorphs, or other reptiles, filling herbivorous roles during the late Early Triassic at Driefontein. Given the rarity of this type of tooth, this may represent a rarity in the paleocommunity. However, no other skeletal material from an azendohsaurid or other herbivorous diapsids have been recovered from the Driefontein locality, making this the first observation of a new kind of diapsid in the ecosystem.

Tooth assemblage descriptions from microvertebrate localities can provide a survey of potential unknown macrovertebrate fauna. These microvertebrate data can also be incorporated with additional bonebed material to create a more complete reconstruction of the paleocommunity by capturing smaller-bodied or rarer taxa [14, 26, 44, 69].

The high overall similarity of the Driefontein teeth examined herein agrees with previous work that the recovery of diverse ecosystems was delayed until the Middle Triassic or possibly even the Late Triassic [9, 14]. Despite phylogenetic evidence indicating that archosaur and other diapsid clades are present in the Early Triassic [17, 70], the ecological expansion of these groups appears to lag. As more microvertebrate tooth assemblages are described throughout the Triassic recovery from the end-Permian extinction, the picture of terrestrial ecosystem recovery will become clearer.

## Conclusions

1. We present the first description of the abundant Driefontein locality diapsid tooth assemblage and a quantitative analysis of the tooth morphotypes.
2. Consistent with the hypothesis of a delayed recovery from the end-Permian mass extinction, the diapsid teeth of the late Early Triassic Driefontein locality seem to retain limited morphological disparity and inferred ecological breadth. This further supports the hypothesis of offset lineage splitting and ecological specialization in archosaurs during the Triassic.
3. Next steps for gauging diapsid diversification and ecological specialization at the Driefontein locality should focus on *in situ* teeth, that is teeth within jaws, to provide more certain taxonomic assignments, and provide estimates for individual variation in tooth morphology as well as test for heterodonty. The use of CT to visualize the morphology of replacement teeth can help in cases where erupted teeth are missing from sockets. Other methods of dietary interpretation for teeth should be used to confirmed ecological interpretations of morphotypes, like OPCR [27], microwear [71], and enamel microstructure [72, 73].
4. The Driefontein locality offers an important window into the terrestrial biotic recovery from the end-Permian mass extinction and our description of multiple tooth morphotypes shows there is a greater diversity of archosauromorphs and other diapsids than are currently known for the locality and for the biozone. Furthermore, microvertebrate tooth assemblages can be used both as a preliminary survey of bonebeds to gauge potential missing diversity and to make ecological inferences about ecosystem paleocommunities [14, 26, 69].

## Supporting information

S1 Dataset Measurements of teeth. (CSV)

S2 Dataset nMDS scorings of teeth. (XLSX)

S1 Questionnaire Inclusivity in global research. (DOCX)
